# A Maximum Muscle Strength Prediction Formula Using Theoretical Grade 3 Muscle Strength Value in Daniels et al.'s Manual Muscle Test, in Consideration of Age: An Investigation of Hip and Knee Joint Flexion and Extension

**DOI:** 10.1155/2017/3985283

**Published:** 2017-01-04

**Authors:** Hideyuki Usa, Masashi Matsumura, Kazuna Ichikawa, Hitoshi Takei

**Affiliations:** ^1^Division of Physical Therapy, Faculty of Health Sciences, Tokyo Metropolitan University, 7-2-10 Higashi-ogu, Arakawa-ku, Tokyo 116-8551, Japan; ^2^Department of Physical Therapy, Faculty of Health Sciences, Kyorin University, 5-4-1 Shimorenjaku, Mitaka-shi, Tokyo 181-8612, Japan; ^3^Department of Physical Therapy, Graduate School of Human Health Sciences, Tokyo Metropolitan University, 7-2-10 Higashi-ogu, Arakawa-ku, Tokyo 116-8551, Japan

## Abstract

This study attempted to develop a formula for predicting maximum muscle strength value for young, middle-aged, and elderly adults using theoretical Grade 3 muscle strength value (moment fair: *M*_*f*_)—the static muscular moment to support a limb segment against gravity—from the manual muscle test by Daniels et al. A total of 130 healthy Japanese individuals divided by age group performed isometric muscle contractions at maximum effort for various movements of hip joint flexion and extension and knee joint flexion and extension, and the accompanying resisting force was measured and maximum muscle strength value (moment max, *M*_*m*_) was calculated. Body weight and limb segment length (thigh and lower leg length) were measured, and *M*_*f*_ was calculated using anthropometric measures and theoretical calculation. There was a linear correlation between *M*_*f*_ and *M*_*m*_ in each of the four movement types in all groups, excepting knee flexion in elderly. However, the formula for predicting maximum muscle strength was not sufficiently compatible in middle-aged and elderly adults, suggesting that the formula obtained in this study is applicable in young adults only.

## 1. Introduction

Evaluation of muscle strength is an important element of the evaluation of patients exhibiting musculoskeletal system or nervous system impairment [1–3]. It is indispensable in clinical evaluations, design and implementation of suitable therapeutic programs, and the prediction of functional ability [4, 5], and so high accuracy and reliability are necessary when performing such an evaluation [6].

Methods for evaluation of muscle strength include manual muscle testing (MMT), methods using an isokinetic dynamometer, and methods for measuring maximum isometric muscle strength using a handheld dynamometer (HHD) [1–4]. MMT does not require a test apparatus and the evaluation is performed via manual resistance imparted by the examiner [5, 6] and so can be performed quickly and easily; for this reason, it is used often in the clinical setting. MMT includes the results of the muscle strength test system that incorporates the effect of gravity, created by Wright and Lovett and amended variously by Lowman, Kendall, and Daniels et al. [7]. In Japan, the MMT method by Daniels et al. [7] is principally used. However, this MMT is not quantitative, as the intervals between grades in the ordinal scale indicated by Grades 0 to 5 (Grade 0: absolutely no visible or palpable activity; Grade 1: some muscle contraction activity visible or palpable; Grade 2: ability to move through the entire range of motion in a position minimizing the effect of gravity; Grade 3: movement through the entire range of motion against only gravity; Grade 4: able to move through the entire range of motion against gravity, but unable to maintain the test position against maximum resistance; and Grade 5: the therapist is unable to break the position maintained by the patient even when imparting maximum resistance) are not equal [7, 8]. Thus, it is a subjective test, and the reliability of detected changes and differences in muscle strength is not high [2–4, 6].

Conversely, muscle strength evaluation methods using an isokinetic dynamometer have high validity and reliability and are considered an objective standard for muscle strength evaluation [4, 9]. However, the apparatuses are inconvenient to use in the clinical setting for several reasons, such as cost, their large size and the subsequent need for a spacious installation location, and their complexity to operate [1–3, 6].

For these reasons, muscle strength evaluation methods that measure maximum isometric muscle strength using HHDs, which are capable of easily making objective and quantitative measurements [1, 3, 5, 9, 10], have been garnering attention in recent years. These methods have the limitation of requiring the joint angle to be fixed but have the advantage of using apparatuses that are lower in cost, smaller, and more easily operated compared to muscle strength evaluation methods that use isokinetic dynamometers [1, 2, 4, 9]. They have also been reported as having good reliability and therefore confirming their usefulness [11–15].

Humans perform physical activities under the influence of gravity, and their activity level is affected by aging, so the maximum muscle strength that an individual is likely to possess depends on their individual characteristics. Thus, in clinical evaluations of muscle strength, which have the objective of designing and evaluating the effectiveness of therapeutic programs suitable for individual patients, it is necessary to take into consideration the characteristics of each patient.

Therefore, in the clinical implementation of a muscle strength evaluation method using an HHD, it is necessary to set the predicted value for the maximum muscle strength, dependent on the characteristics of the subject, as an indicator for the maximum muscle strength that the subject should possess. By comparing the maximum muscle strength value obtained through measurement to the predicted value, the muscle strength evaluation method using an HHD can objectively and quantitatively evaluate muscle strength, taking each subject's individual characteristics into consideration and thereby increasing motivation for exercise drive among subjects. It also gives accurate information on muscle strength to therapists and enables them to create appropriate programs and evaluate their effectiveness.

Although problems have been noted with the objectivity of the MMT grading of Daniels et al., Grade 3 is an exception, as it is an objective baseline of whether it is possible to move completely through the entire range of motion against gravity [7]. Grade 3 muscle strength can be viewed as the static muscular moment needed to support a limb segment against gravity, which is equivalent to the maximal gravitational moment. Thus, the theoretical Grade 3 muscle strength value is calculated based on externally judged criteria by measuring body weight and limb segment lengths and then performing theoretical calculation using anthropometric measures (limb segment weighting factors and center of gravity distance ratio). Therefore, if a relational expression comparing the theoretical Grade 3 muscle strength value and the maximum muscle strength value was known, it would be possible to calculate a predicted value for maximum muscle strength by substituting the calculated theoretical Grade 3 muscle strength value into the expression. As body weight and limb segment length are used in calculating the theoretical Grade 3 muscle strength value, they are dependent on the subject's individual physique; thus, the theoretical Grade 3 muscle strength value can serve as an indicator for the maximum muscle strength that each subject should possess in a relative evaluation of muscle strength values obtained via measurement using an HHD.

In previous studies, with the aim of devising a method using the theoretical Grade 3 muscle strength value from the MMT of Daniels et al. in the prediction of the maximum muscle strength value and elucidating the relationship between the maximum and theoretical Grade 3 muscle strength values, we analyzed four types of arm movements (shoulder flexion, scapular plane elevation and abduction, and elbow joint flexion) and four types of leg movements (hip joint flexion/extension and knee joint flexion/extension) for a total of eight types of movement [16, 17]. After calculating theoretical Grade 3 muscle strength values from body weight and limb segment length for each experimental task, measuring the actual isometric maximum muscle strength using an HHD, and performing a test for noncorrelation and regression analysis, we found a linear relationship between the maximum muscle strength value and the theoretical Grade 3 muscle strength value for each of the eight experimental tasks. It was also suggested that a high degree of precision is possible in the prediction of the maximum muscle strength value outputted from the obtained regression formula. However, these analyses focused exclusively on young adults, and considering the effect of aging on muscle strength [18–25], it has remained unclear whether it is possible to generalize these results from young adults to subjects of different age brackets.

Thus, with the objective of obtaining a formula, similarly to our previous studies [16, 17], for predicting the maximum muscle strength value for flexion/extension of the hip joint and the knee joint from the theoretical Grade 3 muscle strength value according to the MMT grading as described by Daniels et al., this study elucidated the relationship between theoretical Grade 3 muscle strength value and maximum muscle strength value by age group and then investigated differences between the age groups.

## 2. Materials and Methods

### 2.1. Subjects

Subjects were 130 healthy Japanese individuals. The subjects were classified by age (Table 1) into Group A (40 individuals in their 20s and 30s), Group B (46 individuals in their 40s and 50s), and Group C (44 individuals in their 60s and 70s). This study was approved by the Research Safety and Ethics Committee of the Tokyo Metropolitan University, Arakawa Campus (approval number 11038). Prior to the experiments, all subjects were thoroughly informed of the study outline, methods, and the fact that they would not be disadvantaged based on whether they participated in the study. All subjects gave their written consent before participation.

### 2.2. Study Procedure

**Figure 1:**
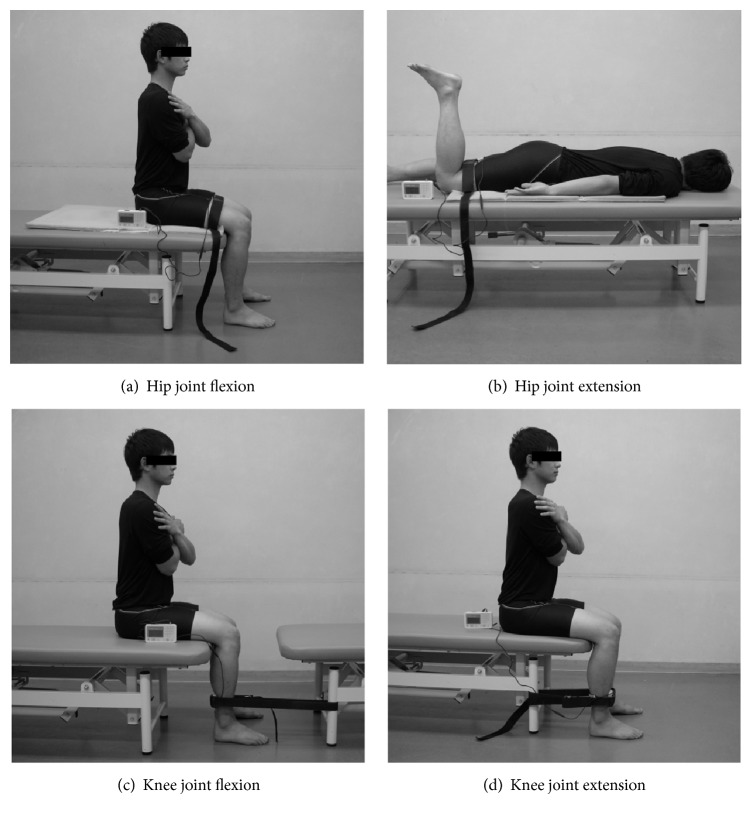
Measurement position.

The experimental tasks were isometric muscle contractions for flexion and extension of the hip joint and for flexion and extension of the knee joint in the dominant leg. The position during the measurement for the hip joint flexion and the knee joint flexion and extension tasks was with the hip and knee joints flexed to 90° in a seated position with the pelvis oriented neutrally and the soles of both feet completely touching the ground. Both arms were folded in front of the torso. The position during the measurement for the hip extension task (hip extension test to isolate the gluteus maximus) was a prone position with the knee joints flexed to 90° and both arms relaxed at the sides of the body (Figure 1) [7].

The handheld dynamometer *µ*Tas MT-1 (ANIMA, Tokyo) pressure sensor was placed at the distal 1/3 position of the thigh during the hip joint flexion/extension tasks and at the distal 1/3 position of the lower leg during the knee joint flexion/extension tasks and affixed to a bed using an inelastic belt and wooden device created by the authors. The thigh length (the distance between the greater trochanter and the knee joint space) and lower leg length (the distance between the knee joint space and the lateral malleolus) necessary for calculating the pressure sensor positioning site were measured before setting the measurement position. From this state, the HHD was zeroed and the maximum muscle strength (Force, *F*) when performing an isometric muscle contraction at maximum effort for each movement was measured. In addition, before measuring *F*, the various movements were practiced and the ability to perform them correctly was confirmed, and sufficient rest was taken to minimize the effect of fatigue. *F* was measured twice for each of the four experimental tasks and the average values were considered as representative values. Body weight, which is necessary for calculating theoretical Grade 3 muscle strength values, was also measured. After performing the measurement, the theoretical Grade 3 muscle strength value (moment fair, or *M*_*f*_) and maximum muscle strength value (moment max, or *M*_*m*_) in Daniels et al.'s MMT were calculated according to the calculation formulas below.

### 2.3. Calculation of *M*_*f*_ and *M*_*m*_

**Figure 2:**
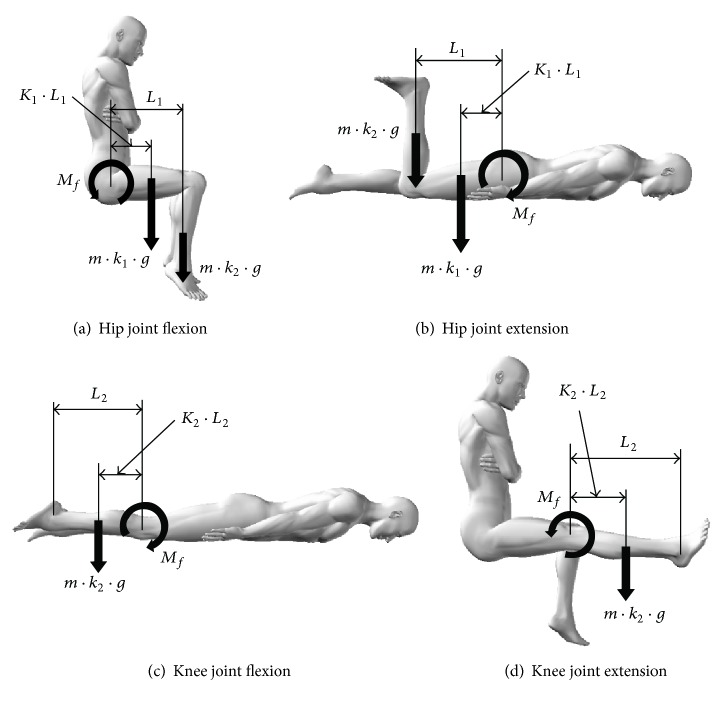
Calculating *M*_*f*_. *m*: body weight (kg), *g*: acceleration due to gravity (m/s^2^) = 9.8, *L*_1_: thigh length (m), *k*_1_: thigh weighting factor (men: 0.1; women: 0.1115), *K*_1_: thigh center of gravity distance ratio = 0.42, *k*_2_: lower leg and foot combined weighting factor (men: 0.0725; women: 0.0685), *K*_2_: lower leg and foot combined center of gravity distance ratio = 0.51, and *L*_2_: lower leg length (m).

In the experimental tasks, *M*_*f*_ (N·m) was calculated from the following formula derived from the balanced relationship of the moment (Figure 2) at the limb position at which the gravitational moment acting on the limb segment is greatest [16, 17, 26, 27].

For hip joint tasks,
  (1) $$\begin{array}{c} M_{f}=m\cdot g\cdot L_{1}\left(\;k_{1}\cdot K_{1}+k_{2}\;\right). \end{array}$$
For knee joint tasks,
  (2) $$\begin{array}{c} M_{f}=m\cdot k_{2}\cdot g\cdot K_{2}\cdot L_{2}, \end{array}$$

where *m* is body weight (kg), *g* is acceleration due to gravity (m/s^2^) = 9.8, *L*_1_ is thigh length (m), *k*_1_ is thigh weighting factor (men: 0.1; women: 0.1115), *K*_1_ is thigh center of gravity distance ratio = 0.42, *k*_2_ is lower leg and foot combined weighting factor (men: 0.0725; women: 0.0685), *K*_2_ is lower leg and foot combined center of gravity distance ratio = 0.51, and *L*_2_ is lower leg length (m).

**Figure 3:**
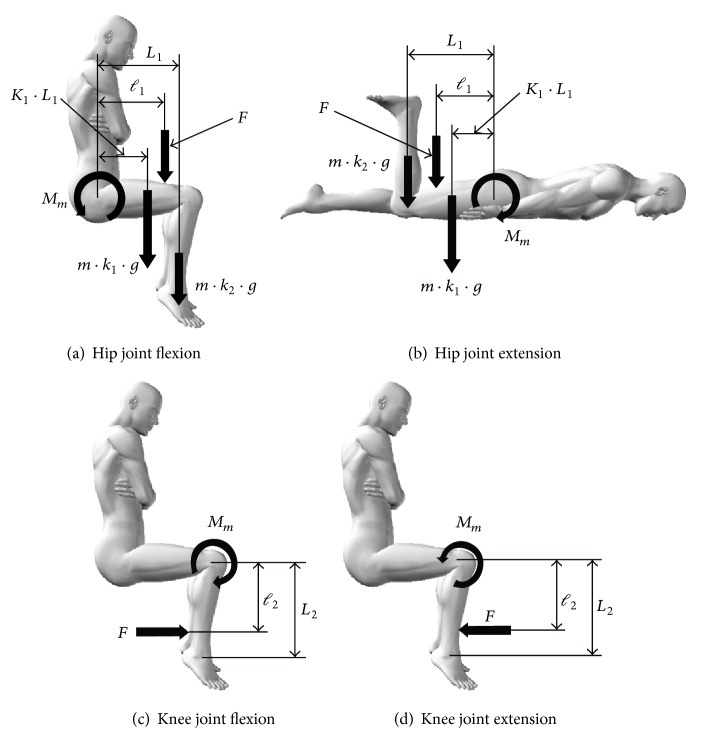
Calculating *M*_*m*_. *m*: body weight (kg), *g*: acceleration due to gravity (m/s^2^) = 9.8, *L*_1_: thigh length (m), *k*_1_: thigh weighting factor (men: 0.1; women: 0.1115), *K*_1_: thigh center of gravity distance ratio = 0.42, *k*_2_: lower leg and foot combined weighting factor (men: 0.0725; women: 0.0685), *L*_2_: lower leg length (m), *ℓ*_1_: distance (m) between the greater trochanter and the measurement site, and *ℓ*_2_: distance (m) between the knee joint space and the measurement site.

*M*_*m*_ (N·m) in each experimental task was calculated using the following formula derived from the balanced relationship of the moment (Figure 3) acting on the limb segment in question at the measurement position for *F*.

For hip joint tasks,
  (3) $$\begin{array}{c} M_{m}=M_{f}+F\cdot l_{1}. \end{array}$$
For knee joint tasks,
  (4) $$\begin{array}{c} M_{m}=F\cdot l_{2}, \end{array}$$

where *F* is maximum resistance (N) during isometric muscle contraction at maximum effort, *ℓ*_1_ is distance (m) between the greater trochanter and the measurement site, and *ℓ*_2_ is distance (m) between the knee joint space and the measurement site.

### 2.4. Statistical Analysis

The statistical software IBM SPSS Statistics Ver. 20 was used for all statistical processing. To investigate the relationship between *M*_*f*_ and *M*_*m*_ for the four experimental tasks by age bracket, an uncorrelated test and a regression analysis were performed for each task and group. To investigate the differences between age brackets in the obtained regression lines for each of the four types of experimental task, covariance analysis of *M*_*m*_ was performed for each experimental task using *M*_*f*_ as the covariant.

## 3. Results

In the hip flexion task, the average proportion of *M*_*f*_ to *M*_*m*_ was 40.7% in Group A, 44.0% in Group B, and 47.8% in Group C; in the hip extension task, 43.8%, 50.9%, and 53.4%; in the knee flexion task, 25.3%, 22.1%, and 40.5%; and in the knee extension task, 13.9%, 17.4%, and 18.4%, respectively.  Table 2 indicates the average of *M*_*f*_ and *M*_*m*_, as well as the proportion of *M*_*f*_ to *M*_*m*_ of the three groups in each experimental task. For each experimental task in Group A, the correlation coefficient was 0.672–0.758, indicating a moderate to strong positive correlation for each task (*p* < 0.05). Similarly, for each experimental task in Group B, the correlation coefficient was 0.486–0.657, indicating a moderate positive correlation for each task (*p* < 0.05). For hip joint flexion/extension and knee joint extension in Group C, the correlation coefficient was 0.376–0.699, indicating a moderate positive correlation (*p* < 0.05), but there was no such correlation found for the knee flexion task (*p* = 0.52).

The results of regression analysis for each task and age bracket except knee joint flexion in Group C (due to the lack of correlation between *M*_*f*_ and *M*_*m*_) are shown in Table 3. All obtained regression lines were useful in predicting *M*_*m*_ using *M*_*f*_  (*p* < 0.05). The coefficients of determination in Groups A, B, and C were 0.452–0.575, 0.236–0.432, and 0.141–0.489, respectively.

No interaction between age group and *M*_*f*_ was observed in the hip joint flexion task (*p* = 0.556) upon a parallelism test in covariance analysis, which confirmed parallelism of the regression lines of each age bracket. A test of the significance of the regression found that the slope of the regression line was nonzero (*p* < 0.001), confirming significance using the covariance. Furthermore, upon testing the difference between tasks, there was a significant difference between Groups A and C (*p* = 0.001). For the hip joint extension test, no interaction between age group and *M*_*f*_  was observed (*p* = 0.299) upon a parallelism test in covariance analysis, which confirmed parallelism of the regression lines of each age bracket. A test of the significance of the regression found that the slope of the regression line was nonzero (*p* < 0.001), confirming significance using the covariance. Upon testing the difference between tasks, there was a significant difference between Groups A and B and between Groups A and C (*p* = 0.007 and *p* < 0.001, resp.). In the knee joint flexion test, no interaction between age group and *M*_*f*_ was observed (*p* = 0.177) upon a parallelism test in covariance analysis between Groups A and B, and the parallelism of the regression lines of each age bracket was confirmed. In a test of the significance of the regression, the slope of the regression line was nonzero (*p* < 0.001), confirming the significance using the covariance. Upon testing the difference between tasks, there was no significant difference between Groups A and B (*p* = 0.101). In the knee joint extension test, an interaction was observed between age group and *M*_*f*_ (*p* = 0.006) upon a parallelism test in covariance analysis, thereby rejecting parallelism of the regression lines for each age group in the knee joint extension task.

## 4. Discussion

In this study, the proportion of *M*_*f*_ to *M*_*m*_ for each of the four experimental tasks in each age bracket was substantially greater than the values in the prior study by Dvir, which examined subjects in their 20s to 40s for hip tasks and subjects in their 30s for knee tasks [28]. This is believed to be caused by differences in restraining the torso and pelvis when measuring *M*_*m*_. In this study, passive restraints for the torso, pelvis, and other sections of the body were not used, and *M*_*m*_ was the maximum muscle strength that could be exerted while the subjects themselves attempted to suppress compensatory actions. In contrast, Dvir measured *M*_*m*_ using an isokinetic dynamometer. The details of the measurement method used by Dvir are not stated, resulting in a lack of clarity. However, passive restraints for the torso or pelvis are commonly used during muscle strength measurements using an isokinetic dynamometer, which is expected to differ significantly from the present study. We believe that the *M*_*f*_ to *M*_*m*_ ratio in this study exhibited a markedly different value because these differences in torso and pelvis restraints have a large effect on the recorded muscle strength values.

In this study, the relationship between *M*_*f*_ and *M*_*m*_, as calculated based on the Grade 3 determination baseline according to the MMT grading by Daniels et al. [7], was investigated for four experimental tasks (hip joint flexion/extension and knee joint flexion/extension) in three age brackets. The results indicated that there was a positive correlation between *M*_*f*_ and *M*_*m*_ for each experimental task and each age bracket except the knee flexion task in Group C, and a linear relationship between *M*_*f*_ and *M*_*m*_ was obtained.

In previous studies [16, 17], we analyzed the relationship between *M*_*f*_ and *M*_*m*_ in arm and leg movements in healthy young adults and found that there was a positive and linear relationship between *M*_*f*_ and *M*_*m*_ in each movement. The results of the present study suggest that the relationship between *M*_*f*_ and *M*_*m*_ is such that there is a linear relationship in the leg exercise of healthy middle-aged and elderly individuals, similarly to muscle strength in young adults. However, this excludes knee flexion in healthy elderly individuals. Furthermore, the results of covariance analysis suggested that this relationship between *M*_*f*_ and *M*_*m*_ differs by age group for hip flexion/extension and knee extension.

Several researchers have reported that the maximum muscle strength of skeletal muscles decreases due to aging [18–25], which is in keeping with our current results. The primary determining factor for the maximum muscle strength of skeletal muscles is muscle mass [29], which is determined by the number of muscle fibers and the average volume (fiber length × fiber cross-sectional area) of the fibers in the skeletal muscle. Changes in muscle mass after adulthood primarily arise through changes in the cross-sectional area of each muscle fiber and through reduction in the number of muscle fibers [30, 31], which progresses alongside aging due to induced apoptosis [32]. Muscle fiber atrophy also occurs due to a breakdown in the balance of muscle protein synthesis and breakdown [33]. These phenomena of aging collectively cause a decrease in muscle mass [19, 23–25, 34, 35], which is the primary age-related factor of differences in maximum muscle strength [19]. For these reasons, the cause of differences between age brackets in the relationship between *M*_*f*_ and *M*_*m*_ in the present study was likely age-related differences in muscle mass. Thus, we believe that the present study successfully verified the relationship between *M*_*f*_ and *M*_*m*_ by age bracket.

However, the results of covariance analysis suggested that the regression lines for Group A and Group B are the same for the hip flexion task and the knee flexion task, but different for the knee extension task. Thus, we believe that which age groups possess different regression lines may depend on the exercise task. Furthermore, the results of the parallelism test in covariance analysis suggest that the slope of the regression line for each age bracket is not the same, due to the interaction seen in the knee extension task. According to Table 3, the slope of the regression line in the knee extension task decreases with increasing age. Thus, the proportion of *M*_*m*_ change resulting from *M*_*f*_ differences also decreases with increasing age. In other words, we believe that the effect of the theoretical Grade 3 muscle strength value—which is dependent on the physique of the individual subject—on the predicted maximum muscle strength value decreases with age among healthy individuals.

However, in all experimental tasks other than knee extension, results suggested that the regression line slopes for each age bracket were equivalent, and so it is reasonable to believe that the exercise task determines whether the degree to which the theoretical Grade 3 muscle strength value affects the predicted maximum strength value will change due to aging.

The coefficients of determination of the obtained regression lines suggested high quality of applying the regression formula in Group A. However, the precision for Groups B and C was not as high as that of Group A. In particular, no correlation was obtained in Group C for the knee flexion task. Therefore, maximum muscle strength can be predicted with great precision using the regression equation in Group A, but not using those in Groups B and C.

Muscle strength is greatest among people in their 20s and 30s and decreases thereafter [31, 36]. These effects were seen in Groups B and C. Changes in the cross-sectional areas of individual muscle fibers and a decrease in the number of fibers cause age-related decreases in muscle strength, but the degree to which those factors are involved is affected by genetics and level of daily physical activity [30], and so significant individual differences are expected. Such individual differences are thought to have affected the precision of predicting the maximum muscle strength values resulting from the regression formulas for Groups B and C. The likelihood of this is also believed to be high due to the higher precision obtained in the group of young adults, who are not yet as affected by aging.

Muscle quality, which is the fraction of adipose tissue and fibrous tissue in a muscle, has been cited as a factor other than muscle mass decrease that contributes to a decrease in muscle strength [37, 38]. Fukumoto et al. [37] cite a decrease in the fraction of type II fibers, which are fast-twitch muscle fibers, a decrease in neurological activity in agonist muscles, and the strength of simultaneous antagonist muscle contraction. Individual differences in these factors could also have affected the precision of predicting the maximum muscle strength value according to the regression formulas for Groups B and C. However, a correlation was not obtained solely for the knee flexion task in Group C, suggesting that exercise task affects the degree to which the aforementioned individual differences in the factor group that contribute to decreasing muscle strength with age will affect maximum muscle strength.

The clinical application of the obtained regression formulas shall be considered. *M*_*f*_  was calculated from formula (1) in the case of hip joint flexion and extension by measuring body weight and thigh length and from formula (2) in the case of knee joint flexion and extension by measuring body weight and lower leg length. For example, in the case of a man with a body weight of 65.0 kg, a thigh length of 0.35 m, and a lower leg length of 0.40 m, the hip flexion/extension *M*_*f*_ would be 25.5 N·m and the knee flexion/extension *M*_*f*_ 9.4 N·m. In the case of a subject in the 20s–30s age bracket, by substituting these values into the obtained regression formulas for Group A, *M*_*m*_ would be predicted to be 62.9 N·m for hip flexion, 58.5 N·m for hip extension, 40.2 N·m for knee flexion, and 73.3 N·m for knee extension.

From the above, the method of predicting maximum muscle strength values using the calculable theoretical Grade 3 muscle strength values in the MMT by Daniels et al. has simplicity for hip joint flexion/extension tasks and knee joint flexion/extension tasks, and so we believe it to be a method that can be easily applied clinically. However, as stated above, the precision of prediction was not high among older individuals, suggesting that the prediction method is applicable in young adults only.

Finally, there are several limitations of the present study. The measured positions for each experimental task in this study were a seated position for the hip joint flexion and knee joint flexion/extension tasks and a prone position for the hip joint extension task. There are cases where subjects are not able to assume a seated or prone position, and in such cases the measurements will be forced to be taken using positions and affixing methods that are different from the present study. However, the tensile force that a muscle exerts differs by muscle length [39]. Consequently, measured values can be affected by the limb position. Passive restraints for the torso and pelvis also affect the measured values. Thus, the predicted values for maximum muscle strength determined using the present results cannot be used in direct comparison against maximum muscle strength measurements obtained when using restraint-utilizing methods or when using limb positions that differ from the measurement limb positions used in the experimental tasks in this study. Additionally, this study concerned only Japanese subjects, so we cannot expect our results to be generalizable to other populations.

## 5. Conclusion

The method of predicting maximum muscle strength values using the theoretical Grade 3 muscle strength values in the MMT by Daniels et al. is simple in hip joint flexion/extension tasks and knee joint flexion/extension tasks, and so we believe it to be a method that can be easily applied clinically. However, prediction formulas varied among different age groups, and the precision was high for young adults but not for middle-aged and elderly adults, suggesting that the formula is applicable only in young adults.

## Figures and Tables

**Table 1:** Subject characteristics.

Group	Number of participants (male, female)	Age^a^	Height (cm)^b^	Weight (kg)^b^	BMI (kg/m^2^)^b^
A	40 (22, 18)	28.3 (20–39)	166.0 (9.0)	60.8 (9.2)	22.0 (2.1)
B	46 (21, 25)	49.8 (40–59)	163.9 (8.1)	62.3 (10.2)	23.1 (3.0)
C	44 (22, 22)	69.6 (61–79)	160.8 (10.2)	58.5 (9.7)	22.6 (2.5)

^a^Age values are mean (minimum–maximum).

^b^Height and weight and BMI values are mean (SD).

**Table 2:** Average of *M*_*f*_ and *M*_*m*_ and ratio of *M*_*f*_ to *M*_*m*_(*M*_*f*_/*M*_*m*_).

Experimental task	Group	*M*_*f*_ (N·m)^a^	*M*_*m*_ (N·m)^a^	*M*_*f*_/*M*_*m*_ (%)
Hip joint flexion	A	25.9 (5.5)	63.7 (17.2)	40.7
	B	26.9 (5.3)	61.1 (17.0)	44.0
	C	25.2 (5.6)	52.7 (14.8)	47.8

Hip joint extension	A	25.9 (5.5)	59.2 (16.5)	43.8
	B	26.9 (5.3)	52.9 (16.4)	50.9
	C	25.2 (5.6)	47.2 (12.4)	53.4

Knee joint flexion	A	8.3 (1.9)	32.8 (18.1)	25.3
	B	8.0 (2.2)	26.2 (17.5)	22.1
	C	7.5 (1.8)	18.5 (9.3)	40.5

Knee joint extension	A	8.3 (1.9)	59.9 (29.0)	13.9
	B	8.0 (2.2)	46.0 (27.2)	17.4
	C	7.5 (1.8)	40.7 (19.7)	18.4

*Notes*. *M*_*f*_: theoretical Grade 3 muscle strength value in the manual muscle test method of Daniels et al.; *M*_*m*_: maximum muscle strength value.

^a^ *M*_*f*_ and *M*_*m*_ values are mean (SD).

**Table 3:** Regression formula and coefficient of determination for each experimental task.

Experimental task	Group	Regression formula	*p* value	Coefficient of determination
Hip joint flexion	A	*M*_*m*_ = 2.364*M*_*f*_ + 2.593	*p* < 0.001	0.574
	B	*M*_*m*_ = 2.111*M*_*f*_ + 4.185	*p* < 0.001	0.432
	C	*M*_*m*_ = 1.855*M*_*f*_ + 5.988	*p* < 0.001	0.489

Hip joint extension	A	*M*_*m*_ = 2.072*M*_*f*_ + 5.673	*p* < 0.001	0.480
	B	*M*_*m*_ = 1.762*M*_*f*_ + 5.470	*p* < 0.001	0.321
	C	*M*_*m*_ = 1.326*M*_*f*_ + 13.816	*p* < 0.001	0.352

Knee joint flexion	A	*M*_*m*_ = 6.482*M*_*f*_ − 20.694	*p* < 0.001	0.452
	B	*M*_*m*_ = 3.939*M*_*f*_ − 5.254	*p* = 0.001	0.236
	C	—	—	—

Knee joint extension	A	*M*_*m*_ = 11.758*M*_*f*_ − 37.261	*p* < 0.001	0.575
	B	*M*_*m*_ = 8.088*M*_*f*_ − 18.703	*p* < 0.001	0.412
	C	*M*_*m*_ = 4.046*M*_*f*_ + 10.465	*p* = 0.012	0.141

*Notes*. *M*_*f*_: theoretical Grade 3 muscle strength value in the manual muscle test method of Daniels et al.; *M*_*m*_: maximum muscle strength value.
